# Barrett’s oesophagus: Is there a need for laparoscopic anti-reflux surgery?

**DOI:** 10.4103/0972-9941.15239

**Published:** 2005-03

**Authors:** Abeezar I. Sarela

**Affiliations:** The General Infirmary at Leeds, The University of Leeds, Leeds LS1 3EX, UK

There has been an alarming increase in the incidence of oesophageal adenocarcinoma in Europe and America and a similar epidemiological trend is likely in Asia. A well-defined morphological sequence in the pathogenesis of oesophageal carcinoma has been recognised, with the serial development of specialised intestinal metaplasia (Barrett’s oesophagus, BE) and progressive grades of dysplasia.^1^ BE is readily recognised by a salmon-pink appearance at endoscopy, with characteristic goblet cells at standard haematoxylin-eosin staining of biopsy specimens, and identifies individuals at increased risk of oesophageal adenocarcinoma.

The predominant aetiological factor for BE is gastroesophageal reflux disease (GORD), wherein the oesophageal mucosa is abnormally exposed to secretions from the stomach (hydrochloric acid) and the duodenum (bile salts). Long-standing GORD is believed to switch the differentiation of pleuripotential oesophageal epithelial stem cells to a BE lineage with subsequent clonal expansion of the novel cell population.^1^ The relative injurious potential of the gastric and duodenal components is vigorously debated and there is no conclusive evidence that one is any worse than the other. It is also argued that bile reflux is more damaging in the alkaline environment that is created by selective control of gastric acid^2^ but there is equal evidence to the contrary.^3^

An important aim in treatment of BE patients is to reduce the risk of oesophageal carcinoma. Proton pump inhibitor (PPI) therapy is highly effective in controlling GORD symptoms for BE patients. However, is symptom-control the optimal clinical end-point for carcinoma-risk reduction? Abnormal acid reflux (oesophageal pH < 4 for longer than 4.5% of monitoring period) or abnormal bile reflux (Bilitec, spectrophotometric absorbance > 0.14 for longer than 1.8% of the monitoring period) persist in up to 50% of patients with long-segment BE, despite good symptom-control with PPI treatment.^4^ Laboratory evidence and limited clinical data suggest that that mere symptom-control is insufficient and objectively confirmed normalisation of acid and bile reflux is important to inhibit carcinogenesis. For example, in BE biopsy specimens, cellular proliferation is significantly reduced and cellular differentiation is increased following normalisation of acid reflux by PPI therapy. In contrast, there is no change in cellular events in patients with persisting abnormal acid reflux.^5^ Eradication of acid reflux can be achieved by serially increasing the PPI dose but this does require repeated pHmetry and is laborious.^6^ PPI therapy does also decrease bile reflux, probably by decreasing the volume of the refluxate, but eradication is unreliable.^4^

Proponents of laparoscopic anti-reflux surgery (LARS) argue superiority over PPI therapy because an operation can restore the physiology of the gastroesophageal junction and provide highly effective control of both acid and bile reflux. Many GORD patients have good symptom-control with PPI but prefer LARS in order to discontinue life-long medication or to obtain relief from intermittent recurrence of heartburn due to inadvertently missing a dose or nocturnal acid breakthrough. Incomplete symptom-control with PPI, pulmonary aspiration and large-volume regurgitation are some other good indications for LARS. Large series of LARS from specialised centres report excellent results and the functional benefits for BE patients appear similar to those for uncomplicated GORD.^7^ The critical issue in promoting and popularising LARS is that the operation is technically demanding as well as uniquely surgeon-dependant and, unlike PPI therapy, the results of major studies cannot be simply generalised. Mature, advanced laparoscopic skills are essential and a definite learning curve for LARS has been recognised.^8^ Peri-operative complications are likely under-reported in the literature and the durability of effectiveness of LARS has been questioned.^9^ Finally, the modified Nissen fundoplication is generally considered to be the standard LARS but experts continue to disagree about several technical issues: What is the place of the posterior partial fundoplication (Toupet) and the anterior partial fundoplication (Watson)? Should the short gastric vessels be routinely divided? How is the short oesophagus recognised pre-operatively and does it require extended trans-mediastinal dissection or gastroplasty? Should prosthesis be used for hiatal closure? Morbid obesity is increasingly prevalent and is an independent risk factor for oesophageal adenocarcinoma. Should a laparoscopic bariatric procedure be the preferred anti-reflux operation for the morbidly obese?

In this issue of *JMAS*, Bamehriz et al report a series of 22 patients with BE and laparoscopic Nissen fundoplication.^10^ The authors are to be commended on meticulous post-operative follow-up by pHmetry and quality of life scores, with excellent functional results. There was complete regression of BE in all cases with BE length < 4cm and it is suggested that surgical therapy may be considered a first-line approach for short-segment BE. Such a recommendation is contentious. It is difficult to definitively evaluate any therapy for cancer-risk reduction in BE because of the paucity of data regarding the final outcome of adenocarcinoma. Changes in length of the BE segment are commonly used as a clinical surrogate for the risk of adenocarcinoma but the biological accuracy of such an approach is undefined. Some other confounding issues are inter-endoscopy and intra-endoscopy variability in length, squamous regeneration over “buried” BE and “pseudo-regression” due to repositioning of the lower oesophagus at anti-reflux surgery. Long-term data are, therefore, important and a recently published, five-year (median) follow-up of a randomised trial of medical therapy versus anti-reflux surgery is intriguing.^11^ Only those patients with *successful* anti-reflux surgery (normal post-operative pHmetry and Bilitec results) remained free of adenocarcinoma and such data strongly support a *well-performed* LARS for BE. It is possible that other trials, without post-operative reflux monitoring, may not have reported superiority of surgery over PPI therapy because of contamination with unsuccessful operations.

So, what is the role of LARS for patients with BE? LARS provides excellent symptom-control and patients with BE should be subject to the same selection criteria as those with uncomplicated GORD. There is a resurgence of interest in anti-neoplastic potential of LARS following the recent, unexpected withdrawal of COX-2 inhibitor drugs, which showed promise as chemo-preventive agents in BE.^12^ A novel clinical trial of PPI therapy versus LARS, with intent to achieve normal pH and Bilitec measurements in both arms and serial evaluation of molecular genetic changes in BE biopsies, has been proposed.^13^ However, until further data are available, cancer-risk reduction should not be the *primary* indication for LARS in BE.

## References

[1] Jankowski JA, Harrison RF, Perry I, Balkwill F, Tselepis C (2000). Barrett’s metaplasia. Lancet.

[2] Kauer WK, Peters JH, DeMeester TR, Ireland AP, Bremner CG, Hagen JA (1995). Mixed reflux of gastric and duodenal juices is more harmful to the esophagus than gastric juice alone. The need for surgical therapy re-emphasized. Ann Surg.

[3] Vaezi MF, Richter JE (1996). Role of acid and duodenogastroesophageal reflux in gastroesophageal reflux disease. Gastroenterology.

[4] Sarela AI, Hick DG, Verbeke CS, Casey JF, Guillou PJ, Clark GW (2004). Persistent acid and bile reflux in asymptomatic patients with Barrett esophagus receiving proton pump inhibitor therapy. Arch Surg.

[5] Ouatu-Lascar R, Fitzgerald RC, Triadafilopoulos G (1999). Differentiation and proliferation in Barrett’s esophagus and the effects of acid suppression. Gastroenterology.

[6] Srinivasan R, Katz PO, Ramakrishnan A, Katzka DA, Vela MF, Castell DO (2001). Maximal acid reflux control for Barrett’s oesophagus: feasible and effective. Aliment Pharmacol Ther.

[7] Yau P, Watson DI, Devitt PG, Game PA, Jamieson GG (2000). Laparoscopic antireflux surgery in the treatment of gastroesophageal reflux in patients with Barrett esophagus. Arch Surg.

[8] Watson DI, Baigrie RJ, Jamieson GG (1996). A learning curve for laparoscopic fundoplication. Definable, avoidable, or a waste of time?. Ann Surg.

[9] Spechler SJ, Lee E, Ahnen D, Goyal RK, Hirano I, Ramirez F (2001). Long-term outcome of medical and surgical therapies for gastroesophageal reflux disease: follow-up of a randomized controlled trial. JAMA.

[10] Bamehriz F, Dutta S, Pottruff CG, Allen CJ, Anvari M (2005). Does laparoscopic Nissen fundoplication prevent the progression of Barrett’s oesophagus?.Is the length of Barrett’s a factor?. J Minimal Access Surg.

[11] Parrilla P, Martinez de Haro LF, Ortiz A, Munitiz V, Molina J, Bermejo J (2003). Long-term results of a randomized prospective study comparing medical and surgical treatment of Barrett’s esophagus. Ann Surg.

[12] Kaur BS, Khamnehei N, Iravani M, Namburu SS, Lin O, Triadafilopoulos G (2002). Rofecoxib inhibits cyclooxygenase 2 expression and activity and reduces cell proliferation in Barrett’s esophagus. Gastroenterology.

[13] Sarela AI, Verbeke CS, Pring C, Guillou PJ (2004). Is symptom control the correct end point for proton pump inhibitor treatment in Barrett’s oesophagus?. Gut.

